# Reference Gene Validation for RT-qPCR, a Note on Different Available Software Packages

**DOI:** 10.1371/journal.pone.0122515

**Published:** 2015-03-31

**Authors:** Ward De Spiegelaere, Jutta Dern-Wieloch, Roswitha Weigel, Valérie Schumacher, Hubert Schorle, Daniel Nettersheim, Martin Bergmann, Ralph Brehm, Sabine Kliesch, Linos Vandekerckhove, Cornelia Fink

**Affiliations:** 1 Ghent University, Department of Internal Medicine, Ghent, Belgium; 2 Justus-Liebig-University, Department of Veterinary Anatomy, Histology and Embryology, Giessen, Germany; 3 Harvard Medical School, Department of Pediatrics, Boston, Massachusetts, United States of America; 4 University of Bonn Medical School, Department of Developmental Pathology, Bonn, Germany; 5 University of Veterinary Medicine Hannover, Institute of Anatomy, Hannover, Germany; 6 University of Münster, Center of Andrology and Reproductive Medicine, Münster, Germany; St. Georges University of London, UNITED KINGDOM

## Abstract

**Background:**

An appropriate normalization strategy is crucial for data analysis from real time reverse transcription polymerase chain reactions (RT-qPCR). It is widely supported to identify and validate stable reference genes, since no single biological gene is stably expressed between cell types or within cells under different conditions. Different algorithms exist to validate optimal reference genes for normalization. Applying human cells, we here compare the three main methods to the online available RefFinder tool that integrates these algorithms along with R-based software packages which include the NormFinder and GeNorm algorithms.

**Results:**

14 candidate reference genes were assessed by RT-qPCR in two sample sets, i.e. a set of samples of human testicular tissue containing carcinoma in situ (CIS), and a set of samples from the human adult Sertoli cell line (FS1) either cultured alone or in co-culture with the seminoma like cell line (TCam-2) or with equine bone marrow derived mesenchymal stem cells (eBM-MSC). Expression stabilities of the reference genes were evaluated using geNorm, NormFinder, and BestKeeper. Similar results were obtained by the three approaches for the most and least stably expressed genes. The R-based packages NormqPCR, SLqPCR and the NormFinder for R script gave identical gene rankings. Interestingly, different outputs were obtained between the original software packages and the RefFinder tool, which is based on raw Cq values for input. When the raw data were reanalysed assuming 100% efficiency for all genes, then the outputs of the original software packages were similar to the RefFinder software, indicating that RefFinder outputs may be biased because PCR efficiencies are not taken into account.

**Conclusions:**

This report shows that assay efficiency is an important parameter for reference gene validation. New software tools that incorporate these algorithms should be carefully validated prior to use.

## Introduction

Reverse transcription quantitative PCR (RT-qPCR) has become the method of choice for quantification of RNA molecules. This powerful tool enables the detection of minimal starting amounts of specific nucleic acids. However, because of its power, this technique is subject to a high degree of technical variation that can severely bias the data. This issue has been thoroughly described and guidelines were recently published that aim to set minimal standards of data reporting to enable an adequate interpretation of published data and provide sufficient details to allow reproduction of experiments by independent researchers [1]. Moreover, these guidelines can help researchers with the set-up of qPCR experiments to conform to the posed guidelines and standardize qPCR experimental set-up. Alas, despite a wide acceptance of these guidelines within the specialized field of qPCR, recent literature reviews indicate that these guidelines have not been sufficiently adopted by a larger group of researchers [2,3].

One of the most crucial points in RT-qPCR data analysis is the choice of a proper normalization method. The parallel quantification of endogenous reference genes is accepted as the most reliable method for normalization of samples [4]. This strategy compensates for most of the variation introduced by pre-PCR and PCR processing. However, in biological systems there are no endogenous reference genes that are truly stably expressed across tissues. Reference gene stability can even vary within the same tissue/cell type between conditions [5,6]. Consequently, the optimal strategy to normalize raw qPCR data is to perform an initial comparison of a set of independently regulated reference genes to assess the most stable ones in each specific experiment or biological setting. This subset of stably expressed reference genes is then used to calculate a normalization factor based on the geometric mean of the most stable reference genes [5,7,8]. Strikingly, this strategy is still not sufficiently applied in the general literature. Recent reviews indicate that in most investigations only one reference gene is used [2,3].

To facilitate the use of multiple reference genes, a series of algorithms were developed to enable a comparison of the stability of the reference genes. Three of these algorithms, i.e. BestKeeper, geNorm, and NormFinder, have been incorporated in free to use Excel based software packages [4,7,9]. The development of these packages, along with newly developed R-based packages using these algorithms has resulted in a rise of research papers in which reference genes are compared. Recently, these algorithms were combined in a free to use web-tool (RefFinder) that, in combination with a fourth comparison termed the comparative CT method [10], enables the assessment of the most stable reference gene. This web-tool uses raw Cq values as raw input and does not enable the input of the specific PCR efficiency of each separate assay. Although this web-tool has been frequently used in the recent literature [11–25], its implementation has not been sufficiently validated to the original described software packages.

In the present paper, we describe the assessment of the most stable reference genes in biopsies of testicular tissue containing carcinoma in situ (CIS), as well as cells from a co-culture model of the human adult Sertoli cell line FS1 and the seminoma-like TCam-2 cells. For reference gene validation, we used the three main algorithms, i.e. BestKeeper, geNorm, and NormFinder and compared these results to the RefFinder output and to the outputs of three R-based software packages incorporating the geNorm and/or NormFinder algorithm.

## Materials and Methods

### Cell lines and cell culture

The human adult Sertoli cell line FS1 [26] was cultured in Medium I (DMEM (Invitrogen, Karlsruhe, Germany), supplemented with 4.5 g/l D-glucose, 1% 4 mM L-glutamine (Sigma, Deisenhofen, Germany), 1% sodium pyruvate (41966, Gipco, by Life Technologies, Eggenstein, Germany), 20% fetal calf serum (PAA, Pasching, Austria), 1% nonessential amino acids, and 1% penicillin/ streptomycin (Biochrom, Berlin, Germany). The human seminoma-like cell line TCam-2 [27] was cultured in Medium II (RPMI 1640 (Gipco) with 2 mM L-glutamine, 10% fetal calf serum, and 1% penicillin/ streptomycin). Each cell line was incubated in 75-cm^2^ flasks (1x10^6^ cells/ flask) at 37°C in a humidified incubator with 5% CO_2_ with medium changes every two or three days. Cells reaching 80% of confluence were used for the further experiments.

### Cell co-culture model

FS1 cells (passages 14–16) were indirectly co-cultured with TCam-2 cells in six well ThinCert (Greiner Bio One, Frickenhausen, Germany) cell culture inserts with translucent membranes and 0.4 μm pores. 50,000 FS1 cells were seeded into the apical chamber on the upper side of the membrane and 50,000 TCam-2 cells were seeded on the underside of the membrane. The cells were co-cultured for 3 weeks in a mixture of 1.5 ml Medium I and 2 ml Medium II at 37°C and 5% CO2 in 2–3 day rotation. One control group consisted of FS1 cells indirectly co-cultured with equine bone marrow derived mesenchymal stromal cells (eBM-MSC). A second control group consisted of FS1 in monoculture. Both control groups were cultured under the same conditions as the experimental group for 3 weeks. Each of the three groups was cultured in four replicates.

### Patient samples

Testicular biopsies from six patients (ages 20–35 years; mean 26 years) were taken under general anaesthesia after written informed consent was obtained. The study was approved by the ethics committee of the Faculty of Medicine of the Justus Liebig University of Giessen (refnr 105/10). Biopsy specimens were cut into two equal pieces. One part was fixed in Bouin solution and embedded in paraffin wax. Sections (5μm) were stained with hematoxylin and eosin and scored according to Bergmann and Kliesch (2010) [28]. Diagnosis of CIS was established by placental alkaline phosphatase immunostaining [28]. Diagnosis of CIS was established by placental alkaline phosphatase immunostaining [29]. The other part was snap-frozen in liquid nitrogen and stored at -80°C until further processing for RT-qPCR.

### RNA isolation and reverse transcription

For RNA isolation, cells were sampled after three weeks of culture in the different conditions. Total RNA was extracted using the peqGold Total RNA kit (Peqlab Biotechnology, Erlangen, Germany) following the kit instructions. From the cryo-preserved biopsies, total RNA was isolated with Trizol (Gibco) according to the manufacturer’s instructions. Quantity and purity (260/280 ratio) of the RNA was assessed by a BioPhotometer (Eppendorf AG, Hamburg, Germany). RNA integrity was assessed by capillary electrophoresis (Bioanalyzer 2100, Agilent Technologies, Boblingen, Germany). DNase digestion was performed incubating 6.65 μl of total RNA (200 ng/μl) with RNase-free DNase I (1 U/μg; Roche, Mannheim, Germany) for 25 min at 37°C, 10 min at 75°C, and cooled at 4°C.

Reverse transcription was performed with the MultiScribe Reverse Transcriptase kit (Applied Biosystems, Darmstadt, Germany) using 0.2 μg RNA in a final volume of 10 μl. Samples were incubated for 25 min at 37°C, 5 min at 75°C and cooled at 4°C.

### RT-qPCR analysis

For RT-qPCR, four replicates of each of the three *in vitro* cell cultures were used along with the six CIS samples. 14 putative reference genes were selected for evaluation of their expression profile (Table 1): TATA box-binding protein (*TBP*), Ubiquitin C (*UBC*); Succinate dehydrogenase, subunit A (*SDHA*), Ras-like protein 13 (*RLP13*), Tyrosine 3-monooxygenase/tryptophan 5-monooxygenase activation protein, zeta polypeptide (*YHWAZ*), Hydroxymethylbilane synthase (*HMBS*), ß2-Microglobulin (*B2M*), Hypoxanthine phosphoribosyltransferase 1 (*HPRT1*), Glyceraldehyde-3-phosphate dehydrogenase (*GAPDH*), Ribosomal protein S18 (*RPS18*, formerly *S18*), ß-actin (*ACTB*), topoisomerase (DNA) II beta (*TOP2B*), insulin-like growth factor 1 receptor (*IGF1R*), and SRY (sex determining region Y)-box 9 (*SOX9*).

**Table 1:** Primers used in the study (Ta: annealing temperature).

Oligo Name	Sequence	direction	Gene symbol	Accession No.	length bp	Ta
GAPDH122-for	aatcccatcaccatcttccag	forward	GAPDH	NM_002046.4	122	59
GAPDH122-rev	aaatgagccccagccttc	reverse				
BACT90-for	ttccttcctgggcatggagt	forward	ACTB	NM_001101.3	89	59
BACT90-rev	tacaggtctttgcggatgtc	reverse				
B2M135-for	ggcattcctgaagctgacag	forward	B2M	NM_004048.2	135	59
B2M135-rev	tggatgacgtgagtaaacctg	reverse				
SDHA85-for	tggttgtctttggtcggg	forward	SDHA	NM_004168.2	85	59
SDHA85-rev	gcgtttggtttaattggaggg	reverse				
UBC74-for	gccttagaaccccagtatcag	forward	UBC	NM_021009.5	74	59
UBC74-rev	aagaaaaccagtgccctagag	reverse				
RLP13-127-for	caaactcatcctcttccccag	forward	RLP13	NM_000977.3	127	59
RLP13-127-rev	ctccttcttatagacgttccgg	reverse				
YHWAZ178-for	atgcaaccaacacatcctatc	forward	YWHAZ	NM 003406.3	178	59
YHWAZ178-rev	gcattattagcgtgctgtctt	reverse				
TOP2B137-for	aactggatgatgctaatgatgct	forward	TOP2B	NM_001068.3	137	59
TOP2B137-rev	tggaaaaactccgtatctgtctc	reverse				
HMBSlOO-for	ctgtttaccaaggagctggaac	forward	HMBS	NM_001258208	100	59
HMBSlOO-rev	tgaagccaggaggaagca	reverse				
S18-88-for	aaaaccaacccggtcagcc	forward	PRS18	X03205	88	59
S18-88-rev	cgatcggcccgaggttatct	reverse				
HPRT94-for	aggaaagcaaagtctgcattgtt	forward	HPRT	NM_000194.	94	59
HPRT94-rev	ggtggagatgatctctcaactttaa	reverse				
TBP-143-for	gagagttctgggattgtaccg	forward	TBP	NM_003194.4	143	59
TBP-143-rev	atcctcatgattaccgcagc	reverse				
SOX9-F	gagcgaggaggacaa	forward	SOX9	NM_000346.3	151	59
SOX9-R	catgaaggcgttcatggc	reverse				
IGF1R-F	tgatgacacggggcgatct	forward	IGF1R	NM_000875	82	59
IGF1R-F	gcttggaggtgctaggactgg	reverse				

All samples were run in triplicate and each run included three no template controls. Standard dilution curves were generated to determine PCR efficiency using cDNA of normal testicular tissue. RT-qPCR was performed in 20 μl final volume containing 1 μl cDNA, 0.6 μl of primers each (10 μM), and 10 μl iQ SYBR Green Supermix (Bio-Rad, Hercules, CA). RT-qPCR was performed on a CFX 96 Real-Time system (Bio-Rad) with a two-step method. The hot start enzyme was activated (95°C for 3 min), and cDNA was amplified for 40 cycles consisting of denaturation at 95°C for 10 s and annealing/extension at 59°C for 30 s. Afterwards a melt curve assay was performed (59°C of 1 min and then the temperature was increased until 94°C by an increment of 0.5°C every 5 s) to detect the formation of non-specifically amplified products.

### Software programs used for statistical analysis and RT-qPCR data processing

Baseline correction and threshold setting were performed using the automatic calculation of the CFX Manager Software (Bio-Rad). Cq determination was performed with the CFX Manager Software (Bio-Rad) using the Single Threshold mode (S1 Table). Linear relative values were assessed by the comparative Ct method ΔCq taking the amplification efficiency (E) into account (S2 Table) and using the sample with the lowest Cq value as reference Cq value (Eq 1). Relative quantities of all samples were assessed by using their validated efficiency into account as well as by assuming 100% overall efficiency, by setting E at 2.

$$\text{RQ}=E^{-\left(minCq-sampleCq\right)}$$ (1)

Both the efficiency adjusted as well as the non-adjusted RQs were used for validation of the most stable reference genes using different algorithms. The geNorm algorithm was used through the originally described geNorm package (Version v3.5) [4], through the R-based NormqPCR package (Version1.8.0) and the SLqPCR package (Version 1.0.0) [30,31]. The NormFinder (Version 0.953) algorithm was used as an Excel add-in and as an R-script (NormFinder for R version 2014-08-23; available at http://moma.dk/normfinder-software)[32] and BestKeeper (Version 1) was used through the BestKeeper software [7]. Finally raw Cq values were used to assess the output of the three software packages using the web-based RefFinder platform (http://www.leonxie.com/referencegene.php).

## Results

### Comparison of geNorm, NormFinder and BestKeeper analysis to different software packages

The ranking of the reference genes was compared between RefFinder and the three different algorithms, geNorm, NormFinder, and BestKeeper, used as described in their original papers. The gene rankings from the original software packages of geNorm and NormFinder differed from their outputs on the RefFinder platform (S1 Document, S3 and S4 Tables). As RefFinder only requires raw Cq values without any possibility to include PCR efficiency, the original geNorm and NormFinder packages were run with relative values, assuming 100% efficiency for all genes. This data was in agreement with the RefFinder output. The raw data output of the BestKeeper software was in agreement with the RefFinder software, regardless of the efficiency used for the reference genes.

The results of the geNorm package were in agreement to that of the geNorm algorithm as provided with the R-based NormqPCR package and the SLqPCR package (data not shown). In addition, the ranking of the genes by the original Excel based NormFinder add-in were similar compared to the NormFinder algorithm within the R-based NormqPCR package and within the NormFinder for R script. However, the measure of the absolute stability value was different for all tested Normfinder applications (S3 and S4 Tables). In concordance to the original geNorm package, the NormqPCR as well as the SLqPCR package provide the pairwise variation of using n/n+1 reference genes to estimate the optimal number of reference genes to be used for data normalization. For the FS1 dataset, the two most stable reference genes can be considered sufficient for normalization based on the 0.15 cut-off for the pairwise variation (S1 Fig). For the CIS data, the optimal number was the three most stable reference genes (S1 Fig). This analysis revealed that the optimal number of reference genes for the FS1 and CIS samples was two and three reference genes respectively (S1 Fig). This data was not provided by the RefFinder output.

### Effect of efficiency adjusted values on NormFinder and geNorm outputs

**Fig 1:**
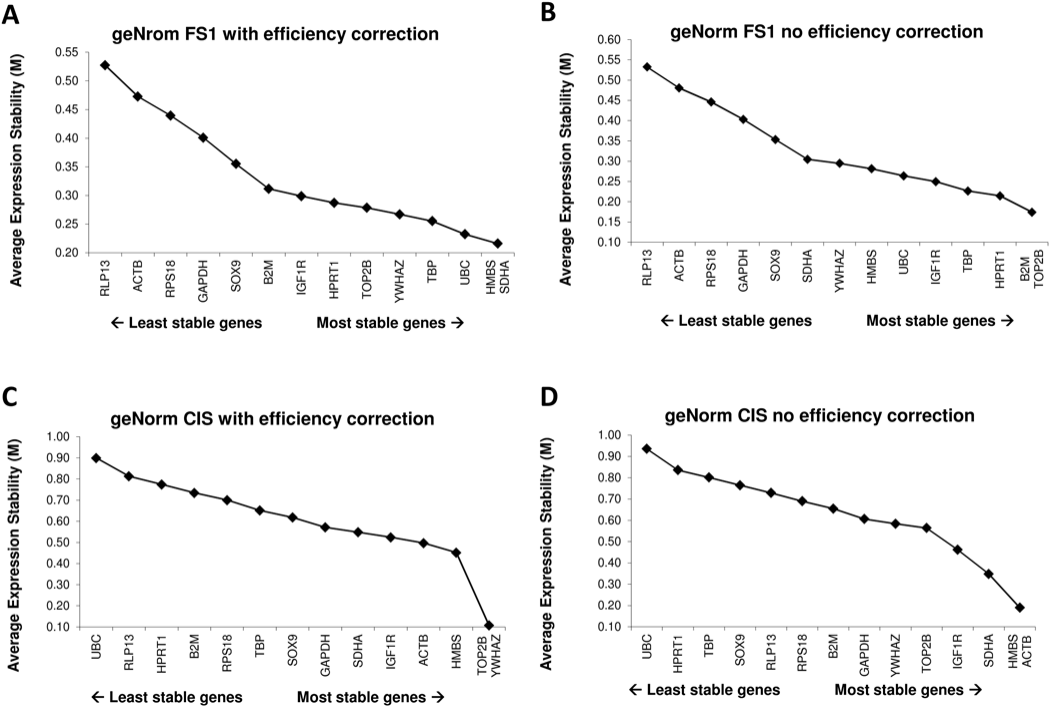
geNorm outputs with efficiency corrected data (A&C) and without efficiency corrected data (B&D) for the two datasets, i.e. FS1 (A&B) and CIS (C&D).

**Fig 2:**
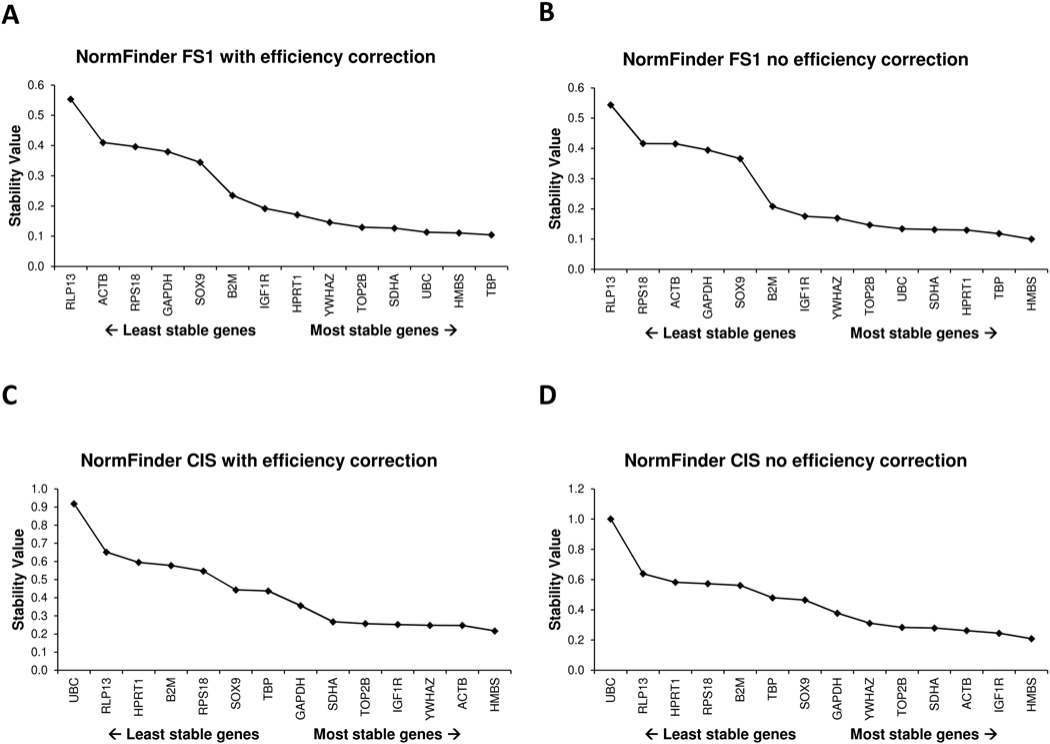
NormFinder outputs with efficiency corrected data (blue bars) and without efficiency corrected data (red bars) for the two datasets, i.e. FS1 (A) and CIS (B).

To assess the influence of using efficiency corrected versus non-corrected values in the validation of reference genes, a comparison of the rankings was made between efficiency corrected and non-corrected relative quantities in sets of samples (Table 2). This comparison revealed that 71% (20/28) and 50% (14/28) of the rankings were different using geNorm (Table 2, Fig 1) and NormFinder (Table 2, Fig 2), respectively. A classification based on minor changes (position switches of one rank up or down) or major changes (position switch of more than one rank up or down) revealed that 16 out of 20 discrepant rankings were major changes with the geNorm algorithm, whereas only 5 out of 14 position changes were major with the NormFinder algorithm. In the FS1 data set the three most stable reference genes as appointed by the geNorm algorithm, SDHA, HMBS and UBC, were replaced by TOP2B, B2M and HPRT1 when gene specific efficiency was not taken into account. Similarly, in the CIS samples, YWHAZ, TOP2B and HMBS would be replaced by ACTB, HMBS and SDHA. The effect on the top three most stable genes with the NormFinder algorithm was less affected by the exclusion of the gene specific efficiency. In the FS1 samples the original top three, HMBS, TBP and UBC, would change to HMBS, TBP and HPRT1 and in the CIS samples the initial top three, HMBS, ACTB and YWHAZ would change to HMBS, IGF1R and ACTB.

**Table 2:** Rankings of the reference genes based on geNorm, NormFinder and BestKeeper, showing both efficiency corrected and non-corrected data used in the original software and the refFinder output for the different algorithms, for the geNorm and NormFinder software.

FS1 samples	geNorm			NormFinder			BestKeeper	
Gene	Efficiency	No efficiency correction	RefFinder	Efficiency corrected	No efficiency correction	RefFinder	Correlation Coefficient	RefFinder
ACTB	13	13	13	13	**12**	**12**	14	**1**
B2M	9	**1/2**	**1/2**	9	9	9	4	**12**
GAPDH	11	11	11	11	11	11	10	**13**
HMBS	1/2	**7**	**7**	1	1	1	5	**3**
HPRT1	7	**3**	**3**	7	**3**	**3**	1	**9**
IGF1R	8	**5**	**5**	8	8	8	6	**8**
RLP13	14	14	14	14	14	14	12	**14**
RPS18	12	12	12	12	**13**	**13**	13	**4**
SDHA	1/2	**9**	**9**	4	4	4	8	**2**
SOX9	10	10	10	10	10	10	11	**10**
TBP	4	4	4	2	2	2	3	**6**
TOP2B	6	**1/2**	**1/2**	5	**6**	**6**	2	**11**
UBC	3	**6**	**6**	3	**5**	**5**	7	7
VWHAZ	5	**8**	**8**	6	**7**	**7**	9	**5**
**CIS samples**	**geNorm**			**NormFinder**			**BestKeeper**	
**Gene**	**Efficiency**	**No efficiency correction**	**RefFinder**	**Efficiency corrected**	**No efficiency correction**	**RefFinder**	**Correlation Coefficient**	**RefFinder**
ACTB	4	**1/2**	**1/2**	2	**3**	**3**	7	**4**
B2M	11	**8**	**8**	11	**10**	**10**	10	**12**
GAPDH	7	7	7	7	7	7	1	**11**
HMBS	3	**1/2**	**1/2**	1	1	1	4	**2**
HPRT1	12	**13**	**13**	12	12	12	14	**5**
IGF1R	5	**4**	**4**	4	**2**	**2**	5	**6**
RLP13	13	**10**	**10**	13	13	13	9	**13**
RPS18	10	**9**	**9**	10	**11**	**11**	12	**3**
SDHA	6	**3**	**3**	6	**4**	**4**	6	**1**
SOX9	8	**11**	**11**	9	**8**	**8**	11	**8**
TBP	9	**12**	**12**	8	**9**	**9**	8	**9**
TOP2B	1/2	**5**	**5**	5	5	5	2	**7**
UBC	14	14	14	14	14	14	13	**14**
YWHAZ	1/2	**6**	**6**	3	**6**	**6**	3	**10**

Switches in ranking from the original algorithm are marked in bold. BestKeeper rankings are based on the correlation coefficient or on the RefFinder output which is only based on the standard deviation of the Cq values.

### BestKeeper ranking

**Fig 3:**
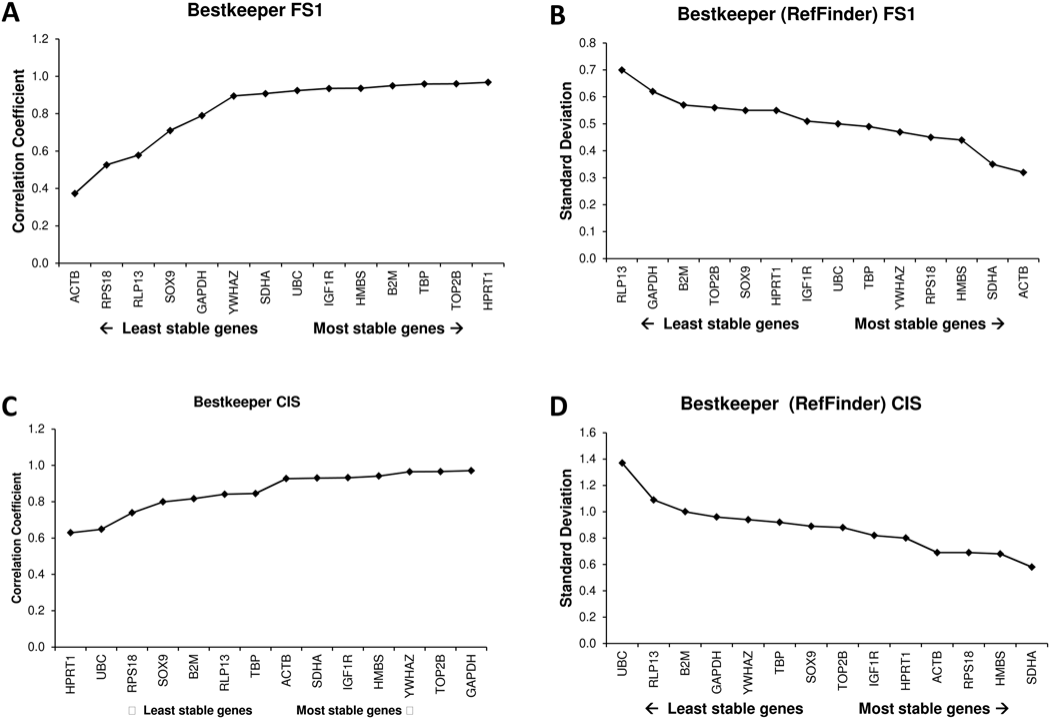
BestKeeper outputs of reference genes ranked by the correlation coefficients (A&C) or by their standard deviation (B&D) for the two datasets, i.e. FS1 (A&B) and CIS (C&D).

The raw data output from the RefFinder platform for the BestKeeper algorithm was equal to the output obtained by the original BestKeeper software. However, the final ranking made by RefFinder was different, because RefFinder ranking is based on the standard deviations of the reference gene Cq-values, whereas the final ranking of the BestKeeper software is usually performed by assessing the correlation coefficients of each individual gene with the geometric mean of all genes (the BestKeeper Index). This different basis for determining gene stability resulted in a substantially different gene ranking (Table 2, Fig 3).

### Comparison of the three algorithms

A comparison of the gene rankings between geNorm, NormFinder, and BestKeeper using efficiency corrected values among the sample sets revealed that in most cases the software packages largely ranked the genes in a similar fashion.

## Discussion

Because of the increasing attention on a proper normalization of qPCR data, there are an increasing number of methods and software packages that have been developed for the validation of the most stabile reference genes. The data of the present manuscript suggests that different software packages should be carefully validated prior to their use in research.

The present paper shows that the use of qPCR efficiency corrected relative values versus non-corrected values impacts the ranking of the reference genes based on their expression stability. Although these differences are in many cases discrete, the use of raw Cq values results in a different set of reference genes that would be selected for normalization. PCR assays with efficiencies varying only slightly from the optimal 100% will have little influence on the final ranking. However, in practice, an optimal PCR efficiency is not always attainable. Here the efficiencies of four assays (RPS18, TBP, UBC and YWHAZ) differed by more than 10% from the optimal 100% efficiency (S2 Table), indicating that efficiency correction in these cases is absolutely necessary.

Interestingly, the geNorm output was more affected by different PCR efficiencies compared to the NormFinder output. The NormFinder software has been described to be less robust with small sample sizes compared to the geNorm algorithm [9]. However, in the present study, the differences between the NormFinder and geNorm algorithm did not vary in the FS1 samples (n = 11) versus the CIS samples (n = 6).

The RefFinder platform is a popular tool for reference gene validation, since it is free and performs a quick analysis using the three most popular algorithms for reference gene validation starting from a single input of the Cq values only. However, despite its application in a set of published studies, this software tool has not been thoroughly validated. Our data shows that the underlying algorithms in the RefFinder software are largely in agreement with the originally described software packages except for one major point. Because of the inability to use gene specific efficiencies, the outputs are based on non-corrected raw Cq values, which biases the gene ranking.

The analysis of the BestKeeper data constitutes an additional concern for the RefFinder software. The BestKeeper software provides two measures that can be used for assessing the stability of the reference genes. The first one is the raw standard deviation of the Cq values, which should be low in the case that an equal amount of input material is used for all samples. These data can be used to exclude specific reference genes when their standard deviation is too high (>1.5). Subsequently, BestKeeper calculates the BestKeeper Index from the geometric mean of the remaining reference genes and performs Pearson correlation of each of the reference genes to the BestKeeper Index to indicate the correlation of that gene with the Index [7]. Hence, the correlation coefficient is a better measure to assess the most stable genes than the standard deviation.

The differences between the underlying algorithms of the three software packages make a direct comparison impossible. Therefore, a mere arithmetic assessment of the most stabile reference genes as performed by the RefFinder platform has no scientific basis. This platform should rather be used as complementary tool to assess reference gene stability, and interpreted by taking their strengths and weaknesses into account. The pairwise correlation of the geNorm algorithm is known to be a strong algorithm for small sample sizes, but is biased towards selecting genes that are mutually correlated (e.g. expressed in the same pathway). The model-based approach by NormFinder has the strength that it can differentiate intragroup variation from intergroup variation and is therefore a suitable tool for identifying candidate genes when different sample groups are to be assessed, yet it requires bigger sample sizes compared to geNorm (>8).

Despite the differences among the three algorithms, the outcome of most stable and least stable reference genes was largely comparable for each sample set, indicating that each software package on its own is capable of differentiating the most stable from the least stable reference genes. Differences in final ranking are mainly observed between genes that do not differ much in stability ranking. This shows that the use of uncorrected data will lead to a suboptimal choice of reference genes, but the risk of using the least stable genes will not arise in any the three software tools.

From the three studied algorithms, only the geNorm algorithm provides a data driven method to define this optimal number based on the pairwise variation obtained by comparing n versus n+1 reference genes. In its original publication, a cut-off of 0.15 was proposed for geNorm, based on the data used in that paper [4]. When the pairwise variation of n versus n+1 reference genes falls below this cut-off, n genes are considered sufficient (2 and 3 for the FS1 and CIS samples respectively). However, it should be noted that this cut-off is a rule of thumb, providing a means to minimize the number of reference genes, while maximizing the stability of the estimated normalization factor. A visual interpretation of the trend of the pairwise variations can also be informative to establish the optimal number of reference genes. In the present project, both sample sets would profit from using more reference genes for normalization, as can be observed from the pairwise variation, which becomes smaller when more reference genes are included (S1 Fig).

The data from the present report also clearly show that different rankings are observed among the two sample sets. Indeed, expression levels of commonly used reference genes are known to vary across different cell or tissue types, and even within one cell or tissue type when subjected to different conditions [5]. Thus, the most stable genes found in the present report cannot be directly applied in other studies using the same cells without prior re-validation of the reference gene stability. Consequently, the current report should be interpreted as a guideline how to perform reference gene validation. Furthermore, it shows that different software packages should be carefully validated prior to their use in research.

## Supporting Information

S1 Document Output from the RefFinder software using the raw Cq values as input data. (DOCX)

S1 Fig Bar plots with pair-wise variations for using n/n+1 reference genes for data normalization to estimate the optimal number of reference genes for normalizing data from the FS1 data set (A) and CIS data set (B). (TIF)

S1 Table Raw Cq values. (XLSX)

S2 Table Efficiencies of the different primer sets. (XLSX)

S3 Table Stability measures from all algorithms for the FS1 samples. (XLSX)

S4 Table Stability measures from all algorithms for the CIS samples. (XLSX)
