# Supplementary material for: Reference Gene Validation for RT-qPCR, a Note on Different Available Software Packages
Source: PLoS One. 2015 Mar 31;10(3):e0122515. doi: 10.1371/journal.pone.0122515 (PMC4380439; doi:10.1371/journal.pone.0122515)

**Supplemental data:**

**RefFinder output for the FS1 samples**

Comprehensive ranking of the FS1 samples by RefFinder, showing the ranking values (table, left) and a graph with these ordered values from most stable to the least stable genes.

| Genes | Geomean of ranking values | |
| --- | --- | --- |
| TBP | 2.63 |  |
| HMBS | 2.82 |  |
| HPRT1 | 3.66 |  |
| TOP2B | 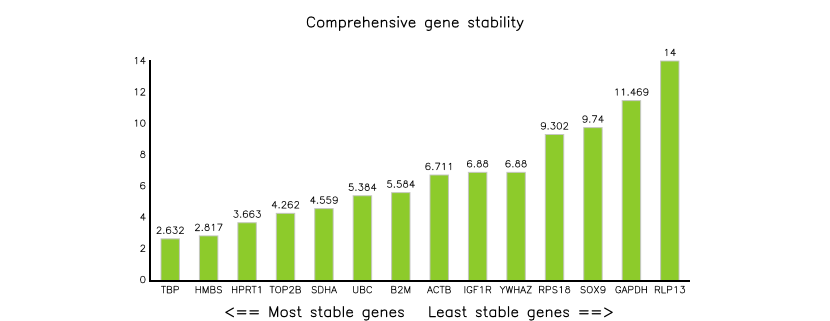4.26 |  |
| SDHA | 4.56 |  |
| UBC | 5.38 |  |
| B2M | 5.58 |  |
| ACTB | 6.71 |  |
| IGF1R | 6.88 |  |
| YWHAZ | 6.88 |  |
| RPS18 | 9.30 |  |
| SOX9 | 9.74 |  |
| GAPDH | 11.47 |  |
| RLP13 | 14.00 |  |

GeNorm ranking of the FS1 samples by RefFinder, showing the ranking values (table, left) and a graph with these ordered values from most stable to the least stable genes.

| Gene name | Stability value |
| --- | --- |
|  |  |
| B2M \| TOP2B | 0.174 |
| HPRT1 | 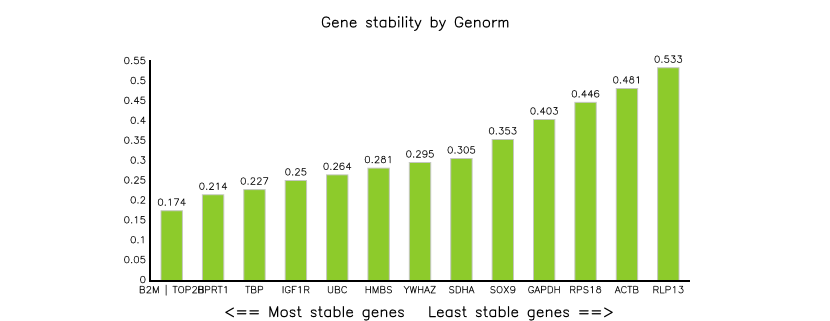0.214 |
| TBP | 0.227 |
| IGF1R | 0.250 |
| UBC | 0.264 |
| HMBS | 0.281 |
| YWHAZ | 0.295 |
| SDHA | 0.305 |
| SOX9 | 0.353 |
| GAPDH | 0.403 |
| RPS18 | 0.446 |
| ACTB | 0.481 |
| RLP13 | 0.533 |

NormFinder ranking of the FS1 samples by RefFinder, showing the ranking values (table, left) and a graph with these ordered values from most stable to the least stable genes.

| Genes | Stability value |
| --- | --- |
|  |  |
| HMBS | 0.144 |
| TBP | 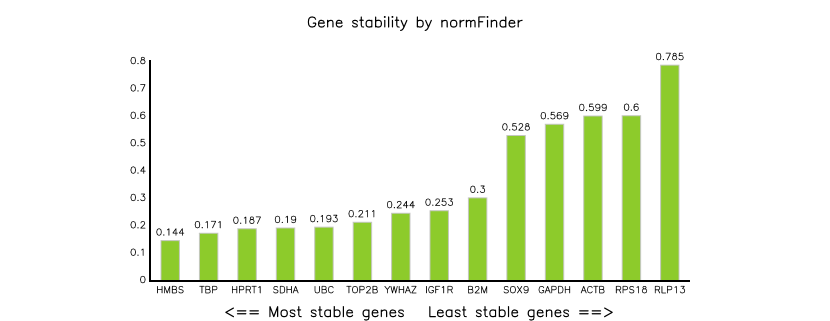0.171 |
| HPRT1 | 0.187 |
| SDHA | 0.190 |
| UBC | 0.193 |
| TOP2B | 0.211 |
| YWHAZ | 0.244 |
| IGF1R | 0.253 |
| B2M | 0.300 |
| SOX9 | 0.528 |
| GAPDH | 0.569 |
| ACTB | 0.599 |
| RPS18 | 0.600 |
| RLP13 | 0.785 |

Bestkeeper ranking of the FS1 samples by RefFinder, showing the output of Bestkeeper with relevant outputs marked in yellow (i.e. standard deviations and coeficients of correlation) and a graph with these ordered values from most stable to the least stable genes which, according to RefFinder is based on the standard deviations alone.

| CP data of housekeeping Genes by BestKeeper | | | | | | | | | | | | | | |
| --- | --- | --- | --- | --- | --- | --- | --- | --- | --- | --- | --- | --- | --- | --- |
|  | B2M | GAPDH | HMBS | HPRT1 | IGF1R | RLP13 | RPS18 | SDHA | SOX9 | ACTB | TBP | TOP2B | UBC | YWHAZ |
| n | 11 | 11 | 11 | 11 | 11 | 11 | 11 | 11 | 11 | 11 | 11 | 11 | 11 | 11 |
| geo Mean [CP] | 21.14 | 19.54 | 26.76 | 26.40 | 27.16 | 24.70 | 10.55 | 25.67 | 31.45 | 18.19 | 27.56 | 26.45 | 29.87 | 22.49 |
| AR Mean [CP] | 21.15 | 19.56 | 26.77 | 26.41 | 27.17 | 24.72 | 10.57 | 25.67 | 31.46 | 18.19 | 27.57 | 26.45 | 29.88 | 22.49 |
| min [CP] | 20.32 | 18.30 | 25.79 | 25.28 | 26.17 | 22.82 | 9.85 | 24.89 | 29.74 | 17.57 | 26.91 | 25.64 | 28.92 | 21.21 |
| max [CP] | 22.64 | 21.05 | 27.54 | 27.54 | 28.26 | 26.07 | 11.48 | 26.22 | 32.26 | 19.01 | 28.69 | 27.51 | 30.68 | 23.63 |
| std dev [+/- CP] | 0.57 | 0.62 | 0.44 | 0.55 | 0.51 | 0.70 | 0.45 | 0.35 | 0.55 | 0.32 | 0.49 | 0.56 | 0.50 | 0.47 |
| CV [% CP] | 2.72 | 3.14 | 1.66 | 2.08 | 1.88 | 2.85 | 4.24 | 1.37 | 1.74 | 1.77 | 1.77 | 2.11 | 1.66 | 2.07 |
| min [x-fold] | -1.77 | -2.36 | -1.96 | -2.18 | -1.98 | -3.69 | -1.63 | -1.72 | -3.28 | -1.53 | -1.56 | -1.74 | -1.93 | -2.41 |
| max [x-fold] | 2.84 | 2.85 | 1.71 | 2.20 | 2.15 | 2.58 | 1.90 | 1.46 | 1.75 | 1.78 | 2.18 | 2.08 | 1.75 | 2.22 |
| std dev [+/- x-fold] | 1.49 | 1.53 | 1.36 | 1.46 | 1.43 | 1.63 | 1.36 | 1.28 | 1.46 | 1.25 | 1.40 | 1.47 | 1.41 | 1.38 |

| Pearson correlation coefficient ( r ) by BestKeeper | | | | | | | | | | | | | | |
| --- | --- | --- | --- | --- | --- | --- | --- | --- | --- | --- | --- | --- | --- | --- |
|  | B2M | GAPDH | HMBS | HPRT1 | IGF1R | RLP13 | SPS18 | SDHA | SOX9 | ACTB | TBP | TOP2B | UBC | YWHAZ |
| GAPDH | 0.760 | - | - | - | - | - | - | - | - | - | - | - | - | - |
| p-value | 0.007 | - | - | - | - | - | - | - | - | - | - | - | - | - |
| HMBS | 0.823 | 0.599 | - | - | - | - | - | - | - | - | - | - | - | - |
| p-value | 0.002 | 0.052 | - | - | - | - | - | - | - | - | - | - | - | - |
| HPRT1 | 0.940 | 0.708 | 0.919 | - | - | - | - | - | - | - | - | - | - | - |
| p-value | 0.001 | 0.015 | 0.001 | - | - | - | - | - | - | - | - | - | - | - |
| IGF1R | 0.907 | 0.700 | 0.870 | 0.903 | - | - | - | - | - | - | - | - | - | - |
| p-value | 0.001 | 0.016 | 0.001 | 0.001 | - | - | - | - | - | - | - | - | - | - |
| RLP13 | 0.456 | 0.120 | 0.694 | 0.635 | 0.439 | - | - | - | - | - | - | - | - | - |
| p-value | 0.158 | 0.725 | 0.018 | 0.036 | 0.177 | - | - | - | - | - | - | - | - | - |
| RPS18 | 0.486 | 0.525 | 0.434 | 0.365 | 0.698 | -0.109 | - | - | - | - | - | - | - | - |
| p-value | 0.130 | 0.097 | 0.183 | 0.269 | 0.017 | 0.750 | - | - | - | - | - | - | - | - |
| SDHA | 0.806 | 0.630 | 0.934 | 0.919 | 0.790 | 0.671 | 0.278 | - | - | - | - | - | - | - |
| p-value | 0.003 | 0.038 | 0.001 | 0.001 | 0.004 | 0.024 | 0.407 | - | - | - | - | - | - | - |
| SOX9 | 0.618 | 0.256 | 0.854 | 0.775 | 0.568 | 0.882 | -0.057 | 0.834 | - | - | - | - | - | - |
| p-value | 0.043 | 0.447 | 0.001 | 0.005 | 0.068 | 0.001 | 0.869 | 0.001 | - | - | - | - | - | - |
| ACTB | 0.349 | 0.747 | 0.186 | 0.250 | 0.168 | -0.072 | 0.220 | 0.192 | -0.030 | - | - | - | - | - |
| p-value | 0.293 | 0.008 | 0.584 | 0.458 | 0.621 | 0.833 | 0.515 | 0.571 | 0.930 | - | - | - | - | - |
| TBP | 0.956 | 0.793 | 0.844 | 0.947 | 0.939 | 0.426 | 0.558 | 0.869 | 0.586 | 0.296 | - | - | - | - |
| p-value | 0.001 | 0.004 | 0.001 | 0.001 | 0.001 | 0.191 | 0.074 | 0.001 | 0.058 | 0.376 | - | - | - | - |
| TOP2B | 0.975 | 0.766 | 0.850 | 0.951 | 0.892 | 0.489 | 0.469 | 0.838 | 0.633 | 0.399 | 0.941 | - | - | - |
| p-value | 0.001 | 0.006 | 0.001 | 0.001 | 0.001 | 0.127 | 0.146 | 0.001 | 0.037 | 0.225 | 0.001 | - | - | - |
| UBC | 0.861 | 0.564 | 0.917 | 0.953 | 0.894 | 0.704 | 0.384 | 0.913 | 0.783 | 0.086 | 0.891 | 0.900 | - | - |
| p-value | 0.001 | 0.071 | 0.001 | 0.001 | 0.001 | 0.016 | 0.244 | 0.001 | 0.004 | 0.802 | 0.001 | 0.001 | - | - |
| YWHAZ | 0.887 | 0.644 | 0.881 | 0.927 | 0.768 | 0.612 | 0.176 | 0.929 | 0.842 | 0.241 | 0.863 | 0.871 | 0.852 | - |
| p-value | 0.001 | 0.033 | 0.001 | 0.001 | 0.006 | 0.045 | 0.604 | 0.001 | 0.001 | 0.475 | 0.001 | 0.001 | 0.001 | - |

| Pearson correlation coefficient ( r ) | | | | | | | | | | | | | | |
| --- | --- | --- | --- | --- | --- | --- | --- | --- | --- | --- | --- | --- | --- | --- |
| BestKeeper vs. | B2M | GAPDH | HMBS | HPRT1 | IGF1R | RLP13 | RPS18 | SDHA | SOX9 | ACTB | TBP | TOP2B | UBC | YWHAZ |
| coeff. of corr. [r] | 0.950 | 0.790 | 0.936 | 0.968 | 0.935 | 0.578 | 0.526 | 0.908 | 0.710 | 0.373 | 0.959 | 0.960 | 0.924 | 0.895 |
| p-value | 0.001 | 0.004 | 0.001 | 0.001 | 0.001 | 0.063 | 0.096 | 0.001 | 0.014 | 0.258 | 0.001 | 0.001 | 0.001 | 0.001 |


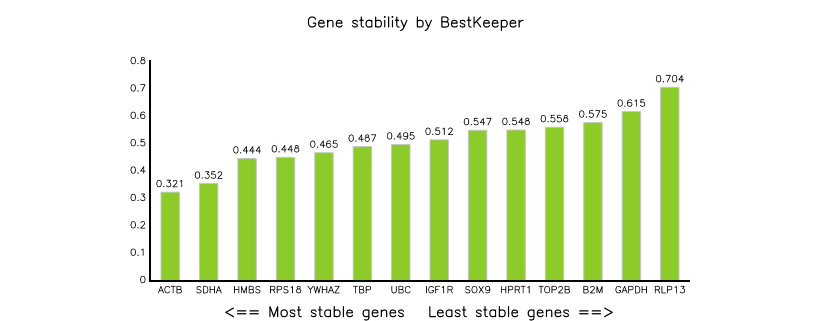


**RefFinder output for the CIS samples**

Comprehensive ranking of the CIS samples by RefFinder, showing the ranking values (table, left) and a graph with these ordered values from most stable to the least stable genes.

| Genes | Geomean of ranking values | |
| --- | --- | --- |
| HMBS | 1.19 |  |
| ACTB | 2.06 |  |
| SDHA | 2.63 |  |
| IGF1R | 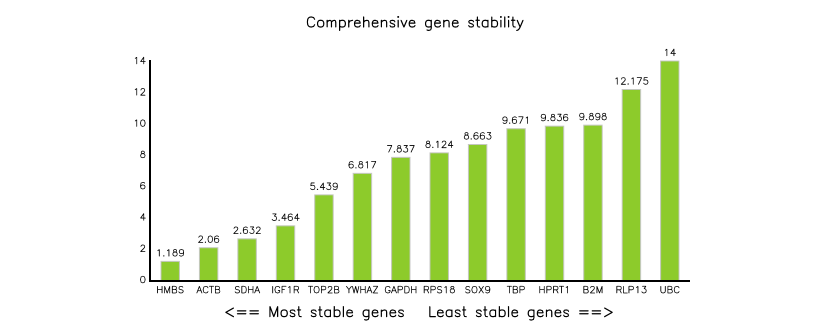3.46 |  |
| TOP2B | 5.44 |  |
| YWHAZ | 6.82 |  |
| GAPDH | 7.84 |  |
| RPS18 | 8.12 |  |
| SOX9 | 8.66 |  |
| TBP | 9.67 |  |
| HPRT1 | 9.84 |  |
| B2M | 9.90 |  |
| RLP13 | 12.17 |  |
| UBC | 14.00 |  |

GeNorm ranking of the CIS samples by RefFinder, showing the ranking values (table, left) and a graph with these ordered values from most stable to the least stable genes.

| Genes | Stability value |
| --- | --- |
|  |  |
| HMBS \| ACTB | 0.190 |
| SDHA | 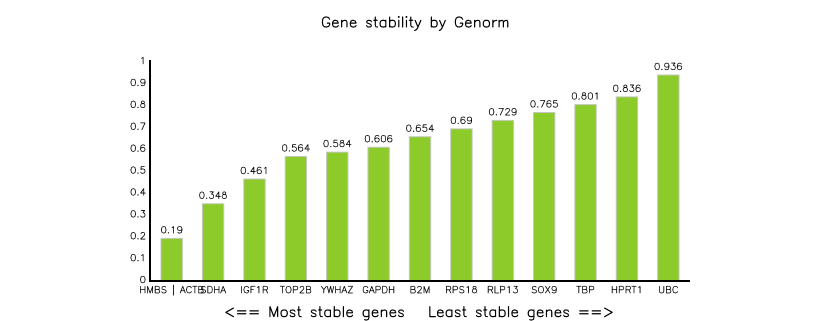0.348 |
| IGF1R | 0.461 |
| TOP2B | 0.564 |
| YWHAZ | 0.584 |
| GAPDH | 0.606 |
| B2M | 0.654 |
| RPS18 | 0.690 |
| RLP13 | 0.729 |
| SOX9 | 0.765 |
| TBP | 0.801 |
| HPRT1 | 0.836 |
| UBC | 0.936 |

NormFinder ranking of the CIS samples by RefFinder, showing the ranking values (table, left) and a graph with these ordered values from most stable to the least stable genes.

| Genes | Stability value |
| --- | --- |
|  |  |
| HMBS | 0.302 |
| IGF1R | 0.354 |
| ACTB | 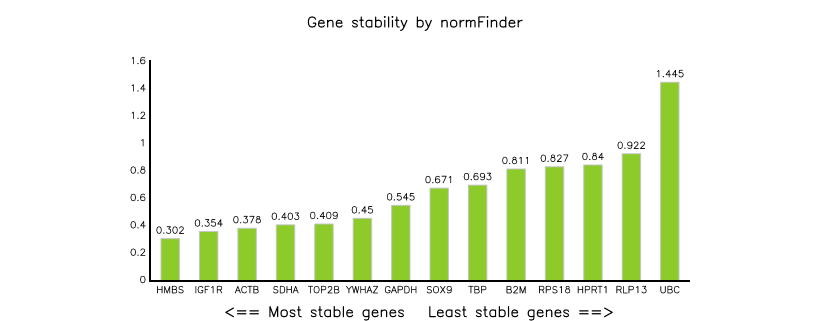0.378 |
| SDHA | 0.403 |
| TOP2B | 0.409 |
| YWHAZ | 0.450 |
| GAPDH | 0.545 |
| SOX9 | 0.671 |
| TBP | 0.693 |
| B2M | 0.811 |
| RPS18 | 0.827 |
| HPRT1 | 0.840 |
| RLP13 | 0.922 |
| UBC | 1.445 |

BestKeeper ranking of the CIS samples by RefFinder, showing the output of BestKeeper with relevant outputs marked in yellow (i.e. standard deviations and coeficients of correlation) and a graph with these ordered values from most stable to the least stable genes which, according to RefFinder is based on the standard deviations alone.

| CP data of housekeeping Genes by BestKeeper | | | | | | | | | | | | | | |
| --- | --- | --- | --- | --- | --- | --- | --- | --- | --- | --- | --- | --- | --- | --- |
|  | B2M | GAPDH | HMBS | HPRT1 | IGF1R | RLP13 | RPS18 | SDHA | SOX9 | ACTB | TBP | UBC | YWHAZ | TOP2B |
| n | 6 | 6 | 6 | 6 | 6 | 6 | 6 | 6 | 6 | 6 | 6 | 6 | 6 | 6 |
| geo Mean [CP] | 20.14 | 21.93 | 27.88 | 27.15 | 25.86 | 22.29 | 10.41 | 24.81 | 25.58 | 19.13 | 26.62 | 28.48 | 23.31 | 26.68 |
| AR Mean [CP] | 20.16 | 21.97 | 27.90 | 27.17 | 25.87 | 22.34 | 10.44 | 24.82 | 25.60 | 19.15 | 26.65 | 28.53 | 23.34 | 26.70 |
| min [CP] | 19.04 | 20.63 | 26.32 | 26.18 | 24.90 | 20.05 | 9.17 | 23.59 | 24.02 | 17.71 | 25.18 | 25.95 | 22.13 | 25.66 |
| max [CP] | 21.86 | 24.59 | 29.44 | 28.31 | 27.51 | 24.20 | 11.84 | 26.19 | 27.00 | 20.45 | 29.06 | 31.28 | 26.04 | 29.34 |
| std dev [+/- CP] | 1.00 | 0.96 | 0.68 | 0.80 | 0.82 | 1.09 | 0.69 | 0.58 | 0.89 | 0.69 | 0.92 | 1.37 | 0.94 | 0.88 |
| CV [% CP] | 4.94 | 4.38 | 2.45 | 2.94 | 3.19 | 4.89 | 6.61 | 2.34 | 3.49 | 3.59 | 3.43 | 4.82 | 4.03 | 3.29 |
| min [x-fold] | -2.14 | -2.47 | -2.94 | -1.96 | -1.94 | -4.74 | -2.36 | -2.32 | -2.96 | -2.68 | -2.73 | -5.75 | -2.27 | -2.02 |
| max [x-fold] | 3.31 | 6.29 | 2.94 | 2.23 | 3.14 | 3.75 | 2.69 | 2.61 | 2.68 | 2.51 | 5.41 | 6.97 | 6.63 | 6.35 |
| std dev [+/- x-fold] | 1.99 | 1.95 | 1.60 | 1.74 | 1.77 | 2.13 | 1.61 | 1.50 | 1.86 | 1.61 | 1.89 | 2.59 | 1.92 | 1.84 |

| Pearson correlation coefficient ( r ) by BestKeeper | | | | | | | | | | | | | | |
| --- | --- | --- | --- | --- | --- | --- | --- | --- | --- | --- | --- | --- | --- | --- |
|  | B2M | GAPDH | HMBS | HPRT1 | IGF1R | RPLP13 | RPS18 | SDHA | SOX9 | ACTB | TBP | UBC | YWHAZ | TOP2B |
| GAPDH | 0.893 | - | - | - | - | - | - | - | - | - | - | - | - | - |
| p-value | 0.016 | - | - | - | - | - | - | - | - | - | - | - | - | - |
| HMBS | 0.794 | 0.875 | - | - | - | - | - | - | - | - | - | - | - | - |
| p-value | 0.059 | 0.023 | - | - | - | - | - | - | - | - | - | - | - | - |
| HPRT1 | 0.155 | 0.462 | 0.568 | - | - | - | - | - | - | - | - | - | - | - |
| p-value | 0.770 | 0.356 | 0.240 | - | - | - | - | - | - | - | - | - | - | - |
| IGF1R | 0.694 | 0.877 | 0.836 | 0.786 | - | - | - | - | - | - | - | - | - | - |
| p-value | 0.126 | 0.022 | 0.038 | 0.064 | - | - | - | - | - | - | - | - | - | - |
| RLP13 | 0.914 | 0.837 | 0.932 | 0.272 | 0.698 | - | - | - | - | - | - | - | - | - |
| p-value | 0.011 | 0.038 | 0.007 | 0.601 | 0.123 | - | - | - | - | - | - | - | - | - |
| RPS18 | 0.908 | 0.865 | 0.577 | 0.140 | 0.680 | 0.676 | - | - | - | - | - | - | - | - |
| p-value | 0.012 | 0.026 | 0.230 | 0.791 | 0.138 | 0.141 | - | - | - | - | - | - | - | - |
| SDHA | 0.838 | 0.941 | 0.908 | 0.370 | 0.796 | 0.883 | 0.723 | - | - | - | - | - | - | - |
| p-value | 0.037 | 0.005 | 0.012 | 0.470 | 0.058 | 0.020 | 0.104 | - | - | - | - | - | - | - |
| SOX9 | 0.439 | 0.662 | 0.803 | 0.855 | 0.897 | 0.607 | 0.321 | 0.695 | - | - | - | - | - | - |
| p-value | 0.384 | 0.152 | 0.054 | 0.030 | 0.015 | 0.201 | 0.534 | 0.125 | - | - | - | - | - | - |
| ACTB | 0.835 | 0.870 | 0.985 | 0.553 | 0.861 | 0.950 | 0.627 | 0.891 | 0.815 | - | - | - | - | - |
| p-value | 0.038 | 0.024 | 0.001 | 0.255 | 0.028 | 0.004 | 0.183 | 0.017 | 0.048 | - | - | - | - | - |
| TBP | 0.405 | 0.760 | 0.743 | 0.769 | 0.796 | 0.485 | 0.396 | 0.736 | 0.766 | 0.661 | - | - | - | - |
| p-value | 0.425 | 0.079 | 0.090 | 0.074 | 0.058 | 0.330 | 0.438 | 0.095 | 0.076 | 0.153 | - | - | - | - |
| UBC | 0.155 | 0.543 | 0.588 | 0.656 | 0.551 | 0.300 | 0.118 | 0.576 | 0.604 | 0.461 | 0.940 | - | - | - |
| p-value | 0.769 | 0.265 | 0.220 | 0.157 | 0.258 | 0.564 | 0.824 | 0.231 | 0.204 | 0.358 | 0.005 | - | - | - |
| YWHAZ | 0.683 | 0.909 | 0.884 | 0.736 | 0.904 | 0.714 | 0.633 | 0.830 | 0.776 | 0.841 | 0.924 | 0.770 | - | - |
| p-value | 0.135 | 0.012 | 0.019 | 0.096 | 0.013 | 0.111 | 0.177 | 0.041 | 0.070 | 0.036 | 0.008 | 0.073 | - | - |
| TOP2B | 0.717 | 0.929 | 0.869 | 0.694 | 0.897 | 0.716 | 0.687 | 0.834 | 0.734 | 0.829 | 0.906 | 0.741 | 0.996 | - |
| p-value | 0.109 | 0.007 | 0.025 | 0.126 | 0.015 | 0.109 | 0.132 | 0.039 | 0.097 | 0.042 | 0.013 | 0.092 | 0.001 | - |

| Pearson correlation coefficient ( r ) | | | | | | | | | | | | | | |
| --- | --- | --- | --- | --- | --- | --- | --- | --- | --- | --- | --- | --- | --- | --- |
| BestKeeper vs. | B2M | GAPDH | HMBS | HPRT1 | IGF1R | RLP13 | RPS18 | SDHA | SOX9 | ACTB | TBP | UBC | YWHAZ | TOP2B |
| coeff. of corr. [r] | 0.817 | 0.971 | 0.941 | 0.629 | 0.932 | 0.841 | 0.739 | 0.930 | 0.799 | 0.927 | 0.845 | 0.648 | 0.965 | 0.966 |
| p-value | 0.047 | 0.001 | 0.005 | 0.181 | 0.007 | 0.036 | 0.094 | 0.007 | 0.057 | 0.008 | 0.034 | 0.164 | 0.002 | 0.002 |


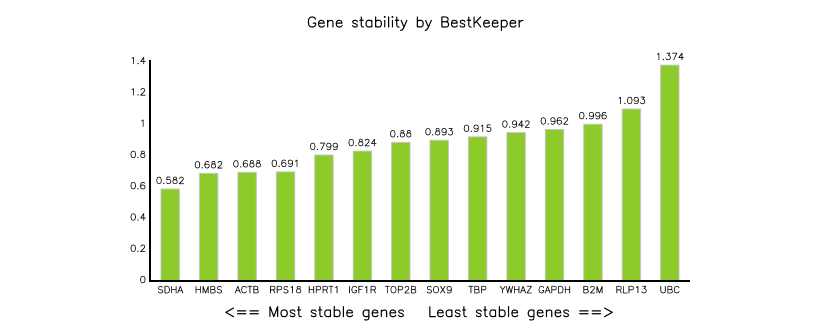

Supplement: S1 Document — (DOCX) [file pone.0122515.s001.docx]
